# CMR adenosine stress perfusion in pediatrics and congenital heart disease: effects on clinical decision making and outcomes

**DOI:** 10.1186/1532-429X-14-S1-O60

**Published:** 2012-02-01

**Authors:** Michael J Campbell, Piers Barker, Brenda Hayes, Raymond J Kim

**Affiliations:** 1 Pediatrics, Duke University Medical Center, Durham, NC, USA; 2 Duke Cardiovascular Magnetic Resonance Center, Duke Unviersity Medical Center, Durham, NC, USA

## Background

In contrast to adults with coronary artery disease (CAD), the use of CMR adenosine stress perfusion in pediatrics and in adults with congenital heart disease (CHD) is not well established. The medical literature reveals an absence of experience evaluating the effects of CMR adenosine stress perfusion on clinical decision making and outcomes in these populations.

### Specific Aims

Evaluate whether CMR adenosine stress perfusion in pediatrics and adults with CHD affects clinical decision making and outcomes.

## Methods

Consecutive patients, who completed CMR adenosine stress perfusion and were < 21yo or > 21yo with CHD, were enrolled. SSFP cine and delayed enhancement CMR (DE-CMR) were performed in a standard manner. Adenosine stress perfusion was performed with administration of adenosine (140 ug/kg/min) for 2-4 minutes and gadolinium (0.1 mmol/kg) using a standard adult protocol. Perfusion defects matching infarct size on DE-CMR and defects corresponding to DE-CMR at the right ventricular insertion site or post-surgical changes were considered negative for ischemia.

## Results

51 studies were performed in 46 patients (mean 25.6 years, 32 < 21 years). Diagnoses and symptoms are listed in Tables 1 and 2. 40/51 studies were negative for ischemia and this finding resulted in no further imaging in 37/40 (93%). 3 patients underwent coronary angiography despite the absence of ischemia on stress perfusion, and none had coronary artery stenosis. 11/51 studies revealed ischemia and 8 were consistent with CAD. 3 patients with ischemia were diagnosed with hypertrophic cardiomyopathy (HCM) based on a) ischemia pattern with small punctate perfusion defects in the mid-portion of the ventricular septum, b) other morphologic findings of HCM. A finding of ischemia led to coronary angiography in 7/10 (1 lost to follow-up). The patients who did not undergo coronary angiography had HCM. 5/7 who underwent coronary angiography had coronary artery stenosis in a pattern consistent with stress perfusion findings. Of these 5 patients, 2 have undergone or are scheduled for coronary artery bypass graft (CABG), 1 is listed for cardiac transplant (pulmonary atresia/intact ventricular septum status post Fontan), 1 is undergoing further workup (myocardial bridge) and 1 was lost to follow-up. Survival rate is 100%. The 3 patients with ischemia in the setting of HCM have been restricted from competitive athletics.

**Table 1:** Diagnoses

Kawasaki disease	9
Tetralogy of Fallot status post repair	6
Hypertrophic cardiomyopathy	4
Suspected hypertrophic cardiomyopathy	4
Anomalous left coronary artery arising from pulmonary artery-repaired	3
Left coronary artery arising from the right coronary sinus-repaired	3
Aortic stenosis status post Ross operation	2
Ventricular septal defect status post repair	2
Coarctation of the aorta status post surgical repair	2
Right coronary artery arising from the left coronary sinus-repaired	2
Anomalous right coronary artery arising from the pulmonary artery-repaired	1
Pseudoxanthoma elasticum	1
Pulmonary atresia with intact ventricular septum	1
Transposition of the great arteries status post arterial switch operation	1
Transposition of the great arteries status post Mustard operation	1
Coarctation of the aorta status post transcatheter stent	1
Bicuspid aortic valve and HTN	1
Right coronary artery aneurysm, etiology unknown	1
Myocardial bridge	1
Scimitar syndrome status post repair and coronary artery bypass graft	1
Congenitally corrected transposition of the great arteries	1
Hypoplastic right pulmonary artery, hypoplastic right coronary artery	1
Dysplastic pulmonary valve with pulmonary insufficiency	1
Sinus venosus atrial septal defect and partial anomalous pulmonary venous return-repaired	1

**Table 2:** Presenting Symptom

Chest pain	19
Previous abnormal diagnostic test	13
Asymptomatic, screening	11
Dyspnea	3
Syncope	3
Nausea and fatigue	1
Irritability	1

## Conclusions

A negative finding on CMR adenosine stress perfusion often results in no further testing, indicating confidence in the result. A positive result can lead to further work-up and positively affect patient outcomes.

## Funding

Institutional.

